# Multi-Step Oxygen Redox Mechanism in the Polyanionic Lithium-Rich Cathode Li_2_FeSiO_4_

**DOI:** 10.1021/jacs.6c02850

**Published:** 2026-09-28

**Authors:** Patrick J. Taylor, Kit McColl, M. Saiful Islam, Benjamin J. Morgan

**Affiliations:** † Department of Chemistry, 1555 University of Bath, Claverton Down, Bath BA2 7AY, U.K.; ‡ 601217 The Faraday Institution, Quad One, Harwell Science and Innovation Campus, Didcot OX11 0RA, U.K.; § Department of Materials, 6396 University of Oxford, Oxford OX2 6NN, U.K.

## Abstract

Lithium-rich cathode materials offer significantly higher energy densities than conventional cathode materials, due to their ability to store charge beyond the formal transition-metal redox limit, via so-called “oxygen redox”. In lithium-rich oxides, oxygen redox is accompanied by molecular O_2_ formation and transition-metal migration, associated with irreversible structural change and poor cycling stability. Lithium-rich silicates provide an alternative lithium-rich chemistry, and have been proposed to be resilient to O_2_ formation at the top of charge, due to their covalently bonded ${\left[{SiO}_{4}\right]}^{4-}$ framework. We report a computational study of the top-of-charge behavior of the prototypical silicate cathode Li_2_FeSiO_4_. Using density functional theory, we predict that fully delithiated FeSiO_4_ is thermodynamically unstable with respect to oxidation of oxide ions to form molecular O_2_ within the bulk material. Ab initio molecular dynamics shows this decomposition occurs spontaneously, via a multistep mechanism involving Fe^IV^ migration and disproportionation to Fe^III^ and Fe^V^, and subsequent Fe^V^ reduction alongside molecular O_2_ formation. Our findings demonstrate that polyanionic frameworks do not inherently prevent O_2_ formation in lithium-rich cathodes, and illustrate how different previously proposed oxygen redox mechanisms can function as steps within a compound process.

## Graphical abstract

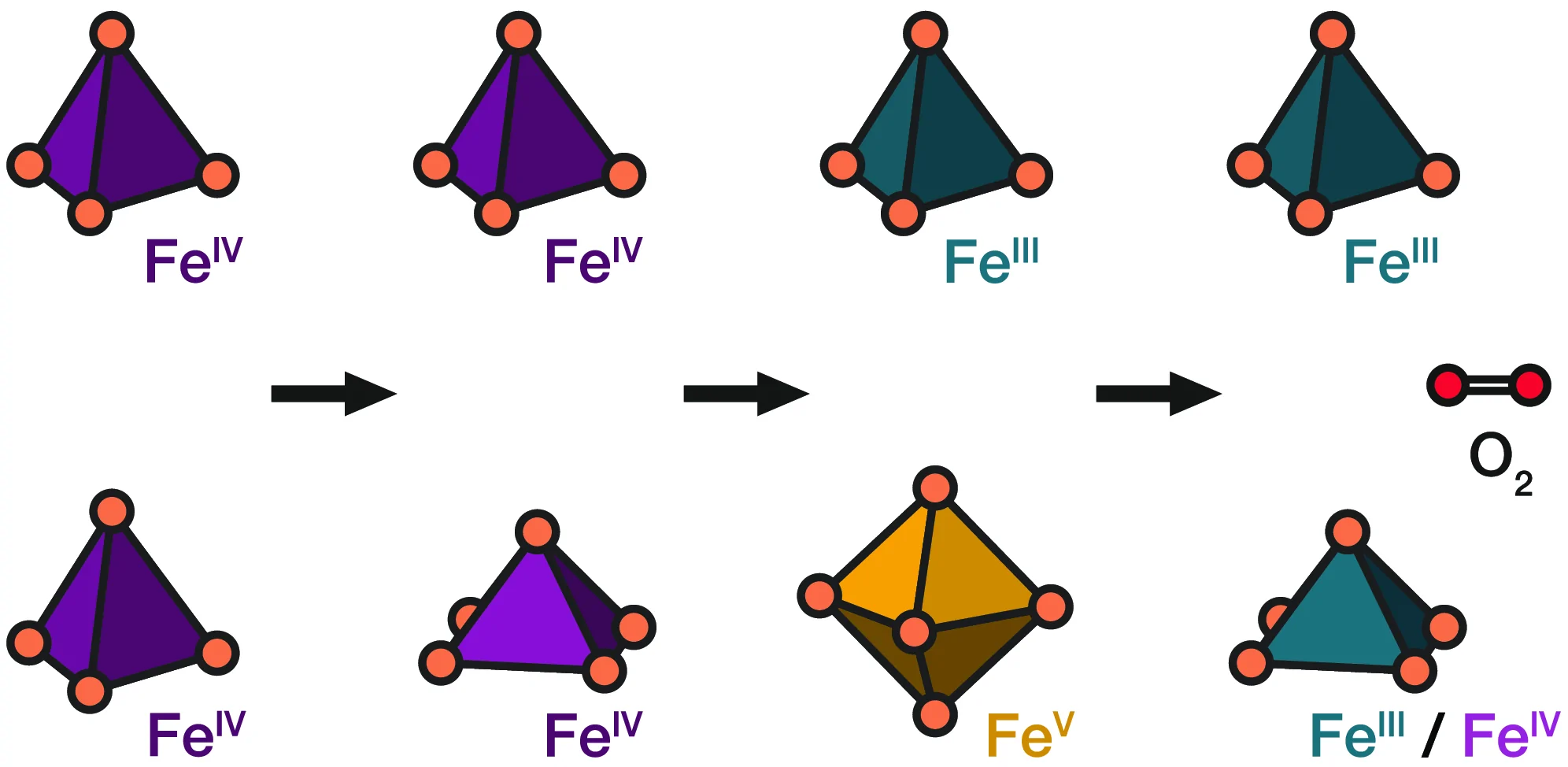

## 1 Introduction

The ongoing global transition to electrified transport is driving increasing demand for lithium-ion batteries with high energy densities.^1^ A significant limiting factor in the energy density of commercial lithium-ion batteries is cathode capacity, which is proportional, via the average operating voltage, to the amount of lithium that can be reversibly intercalated per formula unit during charge and discharge. This direct relationship between lithium content and capacity has focused attention on lithium-rich cathode materials with lithium-to-transition-metal ratios greater than 1, which offer substantially higher theoretical capacities than conventional cathodes.^2–4^

Because lithium-rich materials have more lithium than transition metal per formula unit, complete delithiation formally requires oxidation of the host framework beyond one electron per transition-metal cation. In many cases, this full oxidation exceeds the conventional redox capacity of the component transition-metal cations. The additional capacity is then commonly attributed to formal redox of the anion species, and, as such, is often referred to as “anion redox” or, in the case of lithium-rich oxides and oxyfluorides, as “oxygen redox”.^2,5^

While several classes of lithium-rich cathode materials are known,^6^ research to date has mostly focused on layered lithium-rich oxides, Li_1+*x*_M_*x*_O_2_,^5,7–9^ and lithium-rich disordered rocksalt oxides and oxyfluorides.^10–15^ These oxide and oxyfluoride materials generally exhibit high capacities on first charge, but suffer from hysteresis on their first and subsequent cycles,^16^ plus capacity loss and voltage fade over repeated cycling.^6^ This poor electrochemical reversibility is generally explained as a consequence of the specific oxygen redox mechanisms in these materials;^5,14,16,17^ in many lithium-rich oxides and oxyfluorides, oxygen redox results in the formation of molecular O_2_ ^5,8,9,11,18,19^ and is associated with transition-metal migration and largely irreversible structural rearrangements, which contribute to both hysteresis and voltage and capacity fade.^11,14,16,17,19,20^ This mechanistic link raises a question: might lithium-rich cathodes with alternative anion chemistries exhibit different oxygen redox behaviors and therefore better cycling stability?

One such chemistry is offered by lithium-rich polyanionic cathodes, exemplified by the silicates Li_2_MSiO_4_ (M = Co, Fe, Mn, Ni). These materials have high theoretical capacities, similar to lithium-rich oxides,^21,22^ but markedly different crystal structures. Whereas oxides contain discrete O^2–^ ions, in silicates oxygen is present within polyanionic ${\left[{SiO}_{4}\right]}^{4-}$ tetrahedra, which have strong, highly covalent Si–O bonds that provide silicates with thermal and electrochemical stability.^22–24^ For lithium-rich silicate cathodes specifically, the strong Si–O covalency in Li_2_CoSiO_4_ has been proposed to prevent formation of molecular O_2_.^25^ A second difference lies in the local coordination of oxygen: lithium-rich oxides and oxyfluorides exhibit collinear Li–O–Li units, which give the central oxygen nonbonding 2p states that are readily oxidized,^26^ and which have been identified as a prerequisite for activating lattice oxygen redox in these systems.^27^ In lithium-rich silicates, however, this specific oxygen coordination motif is absent, as oxygen instead bridges between metal centers and silicon. These differences—both the presence of covalently bonded polyanions and the absence of collinear Li–O–Li coordination—suggest that lithium-rich silicates, and other lithium-rich polyanionic cathodes, might not form molecular O_2_ on charging, and could therefore cycle reversibly at high states of charge.

Comparing the oxygen redox behavior of lithium-rich silicates with that of lithium-rich oxides is made more challenging by the range of competing mechanisms that have been proposed for the oxides themselves.^2,5,17,28,29^ These proposed mechanisms include ligand-to-metal charge transfer,^30,31^ formation of localized^32,33^ or delocalized^34,35^ holes on lattice oxygen O^*n*–^ species, oxygen dimerization to form peroxides^36,37^ or molecular O_2_,^11,19^ and high-valent transition-metal oxidation coupled to metal migration.^38,39^ For polyanionic systems, only limited studies have been reported,^25,40–43^ and it remains unknown whether these materials follow any of these proposed oxide patterns or operate through entirely different mechanisms.

Here, we investigate the redox mechanisms of the lithium-rich silicate Li_2_FeSiO_4_. Li_2_FeSiO_4_ has attracted long-standing interest, primarily due to its high theoretical capacity and thermal stability, and the relative abundance of its constituent elements.^21–24,41,44–47^ Li_2_FeSiO_4_ can be delithiated to near-completion, yielding FeSiO_4_,^40^ and gives a charge–voltage profile with two plateau regions and a step at *x* = 1.^45^ Previous DFT calculations predict that the only stable Li_*x*_FeSiO_4_ configurations are the fully lithiated state, Li_2_FeSiO_4_, the 50% delithiated state, LiFeSiO_4_, and the fully delithiated state, FeSiO_4_.^45^ There is consensus that charging from Li_2_FeSiO_4_ to LiFeSiO_4_ (removal of the first lithium per formula unit) is accompanied by oxidation of Fe^II^ to Fe^III^.^23,41,45–48^ There is no consensus, however, as to how to classify the redox process when charging from LiFeSiO_4_ to FeSiO_4_, with both a “high-valent” Fe^III^/Fe^IV^ redox couple^46,47^ and oxygen redox^40,41^ proposed.

To determine the nature of this redox process, we combine static density functional theory (DFT) calculations with ab initio molecular dynamics (AIMD). We find that, in the absence of structural reorganization, removal of the second lithium is charge-compensated by formal oxidation of Fe^III^ to Fe^IV^ with substantial Fe–O covalency. We further predict that fully delithiated FeSiO_4_ is thermodynamically unstable with respect to the formation of molecular O_2_ within the bulk material, despite its polyanionic structure. AIMD simulations reveal a multistep redox mechanism: Fe^IV^ migrates from tetrahedral sites and disproportionates to Fe^III^ and Fe^V^; this Fe^V^ is subsequently reduced as oxygen dimerizes to molecular O_2_.

We therefore conclude that polyanionic frameworks do not inherently prevent O_2_ formation at the top of charge in lithium-rich cathodes. Moreover, we predict that in Li_2_FeSiO_4_, O_2_ formation proceeds via a compound mechanism, comprising multiple processes that have been presented elsewhere as competing oxygen redox mechanisms^2,5,17,28,29^ —​illustrating how such processes can operate together in lithium-rich materials.

## 2 Methods

DFT calculations were performed using the VASP code.^49,50^ The interactions between core and valence electrons were described using the projector augmented-wave (PAW) method,^51,52^ with core configurations of [Ar] for Fe, [Ne] for Si, and [He] for O. For Li, all electrons were treated as valence. These calculations used the r^2^SCAN exchange–correlation functional,^53^ supplemented by the rVV10 van der Waals correction to account for dispersion interactions.^54^ Gaussian thermal smearing was applied with σ = 0.05 eV.

Static calculations and geometry optimizations were performed with a plane-wave cutoff of 650 eV and a Γ-centered ***k***-point mesh with a minimum 0.2 Å^–1^ ***k***-point spacing. Electronic convergence was set to 1 × 10^–6^ eV, while structures were considered converged when all atomic forces were smaller than 0.01 eV Å^–1^. AIMD simulations employed reduced settings, with a plane-wave cutoff of 400 eV, Γ-point only ***k***-point sampling, and electronic convergence set to 1 × 10^–4^ eV. All calculations were spin-polarized with no constraints on magnetic ordering. Initial magnetic moments were set using ferromagnetic ordering for Fe atoms.

AIMD simulations were thermalized for 2 ps in the microcanonical ensemble, with velocities rescaled every 0.1 ps to ramp up the temperature to the desired value. Production simulations were carried out in the canonical ensemble, using a Nosé–Hoover thermostat. AIMD simulations used a 2 fs time step throughout.

Projected densities of states and integrated crystal orbital bond indices (ICOBIs) were computed with the LOBSTER code,^55^ using the pbeVaspFit2015 local basis set to define all projections.^56^

Maximally localized Wannier functions were calculated with Wannier90,^57^ using the selected columns of the density matrix (SCDM) method^58,59^ to obtain initial projections and a convergence criterion of 1 × 10^–10^ Å^2^ applied to the spread functional in all cases. Details of how oxidation states were assigned from the resulting Wannier centers, including the treatment of dimeric species, are given in the Supporting Information (Section S3).

To evaluate the thermodynamic stability of FeSiO_4_, we identified its predicted decomposition products (Fe_2_O_3_, SiO_2_, and O_2_) from the Materials Project database.^60^ We then performed full DFT optimizations on FeSiO_4_ and these competing phases to obtain consistent total energies for calculating the decomposition energy.

The following Python packages were used for workflow automation, data processing, and figure plotting: pymatgen,^61^ ase,^62^ icet,^63^ vasppy,^64^ matplotlib,^65^ scipy,^66^ and numpy.^67^ Structural images were generated using vesta.^68^

## 3 Results and Discussion

### 3.1 Electronic Structure of Li_*x*_FeSiO_4_

To obtain an initial perspective on the redox behavior of Li_2_FeSiO_4_, we examined the electronic structure changes during sequential delithiation. While the lithium-rich silicates exhibit a rich polymorphism, previous studies have found that Li_2_FeSiO_4_ transforms to the inverse *Pmn*2_1_ polymorph after repeated cycling.^45,69^ We therefore adopted this structure (Figure 1) as the starting point for all calculations (see the Supporting Information, Section S1 for further details).

**Figure 1:**
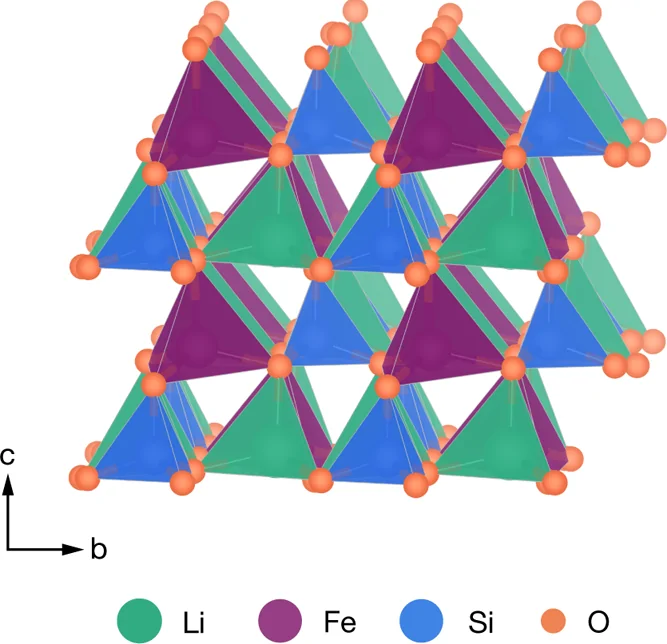
Inverse *Pmn*2_1_ polymorph of Li_2_FeSiO_4_, viewed along the *a* axis. The structure consists of corner-sharing MO_4_ tetrahedra hosted by a slightly distorted hexagonal close-packed array of oxide ions, with zigzag chains of FeO_4_ and SiO_4_ tetrahedra in the *c* direction, alternating with LiO_4_ tetrahedra in the *a* and *b* directions.

We performed DFT geometry optimizations on the three stable configurations of Li_*x*_FeSiO_4_ (*x* = 0, 1, 2; see the Supporting Information, Section S2), modeling delithiation under the assumption of minimal structural rearrangement (i.e., no transition-metal migration). For the 50% delithiated phase, LiFeSiO_4_, we relaxed all symmetry-inequivalent lithium-vacancy orderings in the Li_2_FeSiO_4_ primitive cell (Li_4_­Fe_2_­Si_2_­O_8_), and used the lowest-energy configuration in our subsequent analysis.

Figure 2 shows DFT-calculated projected electronic densities of states (pDOS) plots for Li_2_FeSiO_4_, LiFeSiO_4_, and FeSiO_4_. As an initial assessment of the redox behavior associated with each delithiation step, we focus on the frontier states involved in the corresponding electron removal. For each redox couple, we analyze the highest occupied states in the lithiated phase and the lowest unoccupied states in the delithiated phase. Predominant Fe character in these frontier states suggests a redox process well characterized as cationic redox, while predominant O character suggests anionic redox.

**Figure 2:**
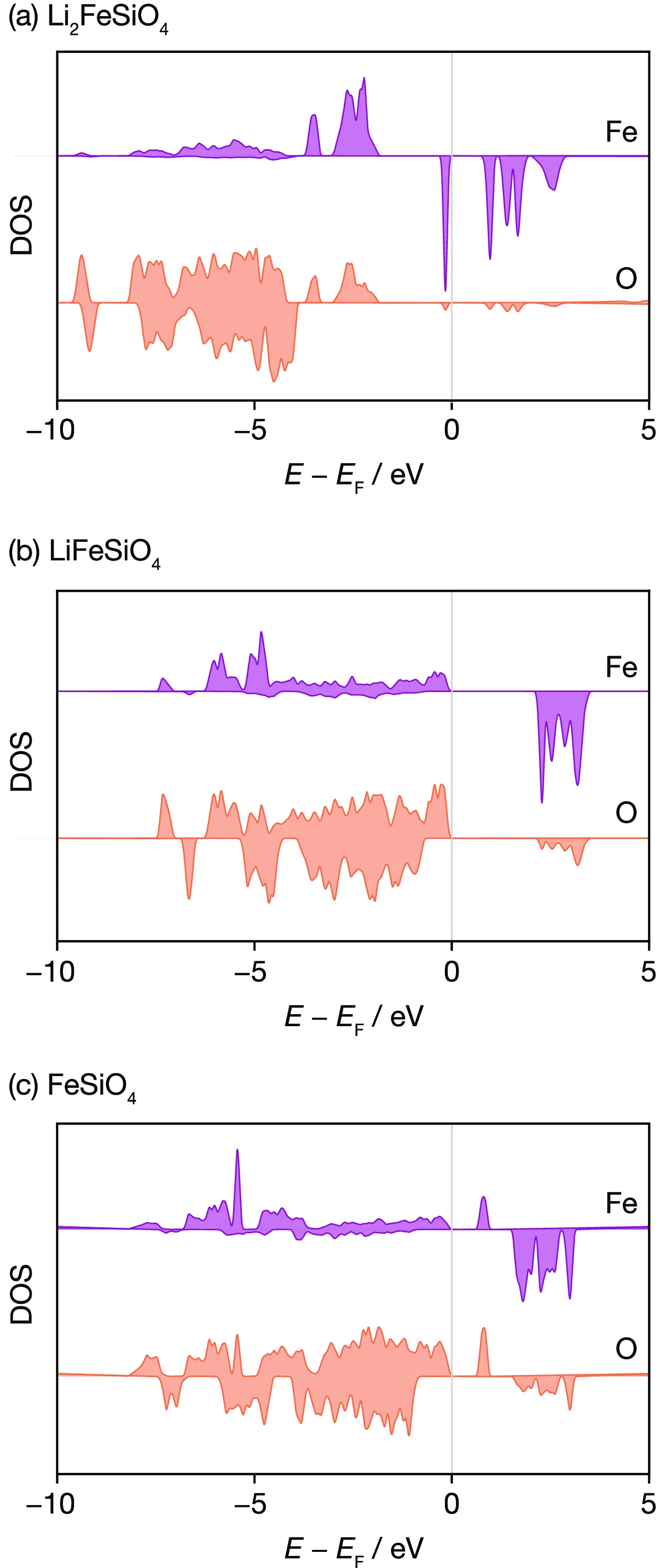
DFT-calculated projected electronic densities of states of (a) Li_2_FeSiO_4_, (b) LiFeSiO_4_, and (c) FeSiO_4_. All energies are relative to the energy of the highest-occupied state for each phase.

For Li_2_FeSiO_4_ (Figure 2a), the Fe states show the characteristic splitting expected for high-spin tetrahedral Fe^II^ (d^6^, 4 unpaired electrons),^70^ with the spin-up states fully occupied and the spin-down states showing ∼1 e occupied. For the first delithiation step (Li_2_FeSiO_4_ → LiFeSiO_4_; Figure 2b), the occupied spin-down Fe state shifts across the Fermi level, consistent with oxidation to high-spin Fe^III^ (d^5^). Based on the predominant Fe character of these frontier states and the alignment with a textbook d^6^ → d^5^ process, we characterize this first delithiation as conventional cationic redox, consistent with previous experimental and computational studies.^45,48,71^

The second delithiation from LiFeSiO_4_ to FeSiO_4_ (Figure 2c) presents a more complex picture. The highest occupied states in LiFeSiO_4_ and the lowest unoccupied states in FeSiO_4_ both show substantial Fe–O hybridization. In contrast to the first delithiation, electrons are removed from mixed Fe–O states rather than strongly Fe- or O-dominated orbitals, making it impossible from the pDOS data alone to classify this redox process as either purely cationic or purely anionic.

To assign Fe and O oxidation states, and to quantify the contribution of each species to the second redox process, we applied three further analyses: Fe magnetic moments, integrated crystal orbital bond indices (ICOBIs), and Wannier analysis.

Fe magnetic moments are correlated with the number of unpaired electrons formally assigned to each Fe center, and can be used as a proxy for oxidation state.^72,73^ Given the tetrahedral coordination of Fe and weak-field oxygen ligands, we would expect Fe^II^ and Fe^IV^ to have 4 unpaired electrons and Fe^III^ to have 5 unpaired electrons. Fe^II^ and Fe^IV^ can be distinguished based on the state of charge.

Figure 3a shows calculated Fe magnetic moments for our geometry-optimized structures for Li_2_FeSiO_4_, LiFeSiO_4_, and FeSiO_4_. For Li_2_FeSiO_4_, we find a magnetic moment of 3.57 μ_B_, consistent with high-spin tetrahedral Fe^II^. With removal of the first lithium to form LiFeSiO_4_, the magnetic moment increases to 4.06 μ_B_, consistent with oxidation to Fe^III^. For fully delithiated FeSiO_4_, the magnetic moment then decreases to 3.29 μ_B_, consistent with Fe^IV^. These magnetic moments are consistent with an assignment of the full redox process as formal oxidation of Fe from Fe^II^ through Fe^III^ to Fe^IV^.

**Figure 3:**
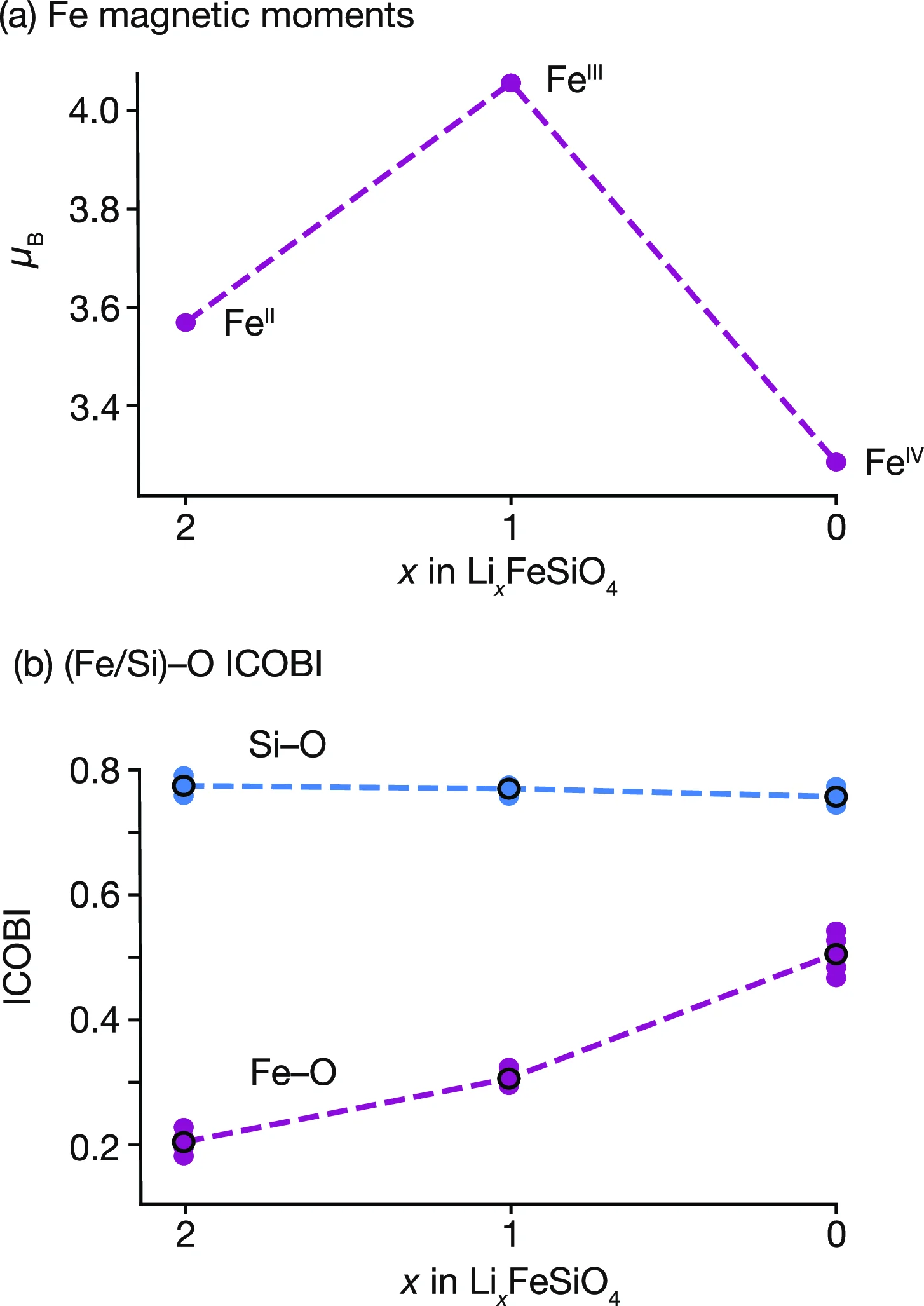
(a) Fe magnetic moments, and (b) Fe–O and Si–O ICOBIs at *x* = 0, 1, 2. Mean values in (b) are indicated by points with a black edge.

While Fe magnetic moments can be used to assign formal Fe oxidation states, they provide no information about the degree to which oxygen participates in these redox processes. ICOBIs provide a quantitative description of Fe–O covalency, which can be interpreted as a measure of oxygen participation in each redox couple. The crystal orbital bond index is the density of states weighted by the density matrix expressed in a local basis,^74^ and the integral of this quantity up to the Fermi level (the ICOBI) corresponds to the classical notion of bond order: 0 for purely ionic bonds, 1 for single covalent bonds, and 2 for double bonds. For purely cationic redox, we would expect minimal Fe–O covalency throughout delithiation (the ionic limit is given by ICOBI ≈ 0), while substantial covalency (ICOBI ≫ 0) indicates joint Fe/O participation in the redox process.

Figure 3b shows calculated Fe–O ICOBIs for the three geometry-optimized structures. For Li_2_FeSiO_4_, the mean Fe–O ICOBI is 0.20, corresponding to highly ionic Fe–O bonding. Removal of the first Li to form LiFeSiO_4_ increases the Fe–O ICOBI to 0.31, corresponding to only a moderate increase in Fe–O covalency. Removal of the second Li, however, increases the ICOBI to 0.51, indicating considerable Fe–O covalency in the fully delithiated end member despite the formal assignment of Fe^IV^ from the magnetic moment data. For comparison, the Si–O ICOBIs (also shown in Figure 3) are approximately constant at ∼0.77 across all three compositions. The Fe–O ICOBI of 0.51 in FeSiO_4_ is thus two-thirds that of the strongly covalent Si–O bonds within the polyanionic SiO_4_ units, highlighting the extent of Fe–O covalency in the fully delithiated material.

A complementary perspective on the degree of Fe–O covalency and its evolution under delithiation is provided by Wannier analysis. Transforming our plane-wave DFT calculations into spatially localized Wannier functions provides a more chemically intuitive representation of the electronic structure. Visualizing the isosurfaces for individual Wannier functions reveals the degree of their Fe versus O character, while associating each Wannier center with its closest atom enables integer electron counting for each atom and thus unambiguous assignment of formal oxidation states.

Figure 4 shows Wannier isosurfaces for the lowest-energy unoccupied state for Li_2_FeSiO_4_, LiFeSiO_4_, and FeSiO_4_. For Li_2_FeSiO_4_, this isosurface is *d*_*z* ^2^_-like and is centered on Fe, with minimal oxygen contribution, consistent with ionic Fe^II^. For LiFeSiO_4_, small contributions from neighboring O become visible, consistent with the increased Fe–O ICOBI at this composition. For FeSiO_4_, the isosurface is qualitatively different: substantial density extends across multiple oxygen atoms, including some not directly bonded to the central Fe. This progression from localized Fe d orbitals to highly delocalized Fe–O states provides further evidence for the transition from highly ionic to more covalent bonding identified by the ICOBI analysis. While the Wannier isosurfaces show a clear increase in Fe–O delocalization, electron counting from Wannier centers confirms the oxidation-state assignments suggested by magnetic moments (see the Supporting Information, Section S3): this assigns 6 d electrons per Fe in Li_2_FeSiO_4_ (Fe^II^), 5 d electrons in LiFeSiO_4_ (Fe^III^), and 4 d electrons in FeSiO_4_ (Fe^IV^).

**Figure 4:**
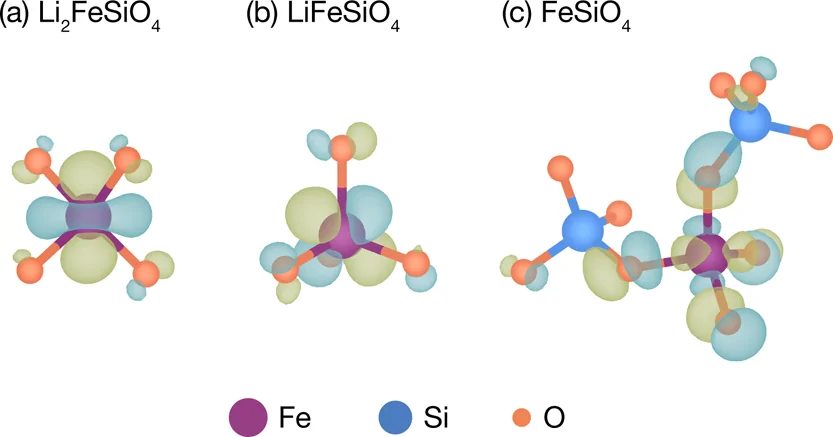
Wannier isosurfaces (isovalue ± 1.5 Å^–3/2^) of the lowest-energy unoccupied state at *x* = 0, 1, 2; green and cyan surfaces correspond to positive and negative isovalues, respectively. Additional atoms are shown in (c) to illustrate the extent of the isosurface, which extends to peripheral O atoms not directly bonded to the central Fe.

These three complementary analyses yield a consistent description in which the two delithiation steps in Li_2_FeSiO_4_ show fundamentally different redox behavior. The first delithiation is well described as conventional cationic redox, i.e., formal Fe^II^ → Fe^III^ oxidation with minimal change in Fe–O covalency. The second delithiation, while formally assigned as Fe^III^ → Fe^IV^ based on magnetic moments and Wannier electron counting, yields an end product (FeSiO_4_) with substantial Fe–O covalency and Fe-centered hole states that are extensively delocalized over nearby oxygen centers. Thus, the second redox process has mixed cationic/anionic character, with significant electron density removed from oxygen atoms alongside the formal Fe oxidation.

### 3.2 Thermodynamic Stability of FeSiO_4_

The electronic structure analysis above is based on geometry optimization of the unreorganized FeSiO_4_ structure at the top of charge, which relaxes to a local potential energy minimum in which delithiation has occurred without Fe or Si migration. In other lithium-rich systems (both layered oxides and disordered rocksalts), direct DFT relaxation of top-of-charge structures often gives an incomplete description of redox behavior. Such systems are generally thermodynamically unstable with respect to the formation of metal-rich regions and molecular O_2_ and, in these cases, molecular dynamics simulations starting from geometry-optimized structures produce spontaneous energy-lowering structural reorganizations, involving transition-metal migration and O–O dimerization.^9,11,14^ In these lithium-rich oxides and oxyfluorides, such structural reorganization provides an explanation for experimentally observed voltage hysteresis over their first charge–discharge cycle.^16,19^ Li_2_FeSiO_4_ exhibits similar first-cycle voltage hysteresis, suggesting that it too undergoes some degree of structural reorganization upon delithiation, particularly when charged beyond LiFeSiO_4_.^47^

To assess whether FeSiO_4_ is similarly thermodynamically unstable, we calculated its stability with respect to decomposition into competing phases (see Methods for details). These calculations predict that FeSiO_4_ is unstable by 0.23 eV/atom with respect to decomposition via

1 $${FeSiO}_{4}\to \frac{1}{2}{Fe}_{2}O_{3}+{SiO}_{2}+\frac{1}{4}O_{2}$$

This reaction corresponds to the formal reduction of Fe^IV^ (FeSiO_4_) to Fe^III^ (Fe_2_O_3_) and the concomitant oxidation of O^II–^ to O^0^ (molecular O_2_). Our thermodynamic analysis, therefore, predicts that FeSiO_4_ is unstable with respect to formation of molecular O_2_, which may be released as a gas or, as proposed for lithium-rich layered^9,19^ and disordered rocksalt systems,^11,14^ remain trapped in the bulk. This prediction is consistent with the broader instability of charged oxide cathodes with respect to decomposition and oxygen release.^75,76^

### 3.3 Kinetic Stability of FeSiO_4_

While our thermodynamic analysis predicts that FeSiO_4_ is unstable with respect to decomposition into Fe_2_O_3_, SiO_2_, and O_2_, it does not indicate whether this transformation is kinetically accessible or the mechanism by which it occurs. As noted above, AIMD simulations of other lithium-rich systems have revealed rapid structural reorganization involving transition-metal migration and O–O bond formation.^9,11,14^ To investigate whether FeSiO_4_ exhibits similar kinetic instability, we performed AIMD simulations at 500 and 1000 K, starting from an optimized 2 × 3 × 3 expansion (216 atoms) of our FeSiO_4_ structure. The elevated temperatures accelerate sampling of the local potential energy surface, allowing our simulations to sample barrier-crossing events that would be too infrequent to observe on AIMD time scales at lower temperatures.

Figure 5 shows the total energy evolution during the AIMD simulations at 500 and 1000 K. These trajectories both show substantial energy decreases of ∼8 eV and ∼12 eV respectively, indicating significant structural reorganization on the picosecond time scale. To characterize these structural changes, we selected four MD frames from each trajectory and relaxed them to local minima using DFT geometry optimization. The relaxed structures lie 8.55 eV (500 K) and 16.79 eV (1000 K) below unreorganized FeSiO_4_; these are total energies for the 216-atom supercell, and the difference between them reflects the extent of reorganization reached in each trajectory. The lowest-energy relaxed structure is obtained from frame iv of the 1000 K simulation.

**Figure 5:**
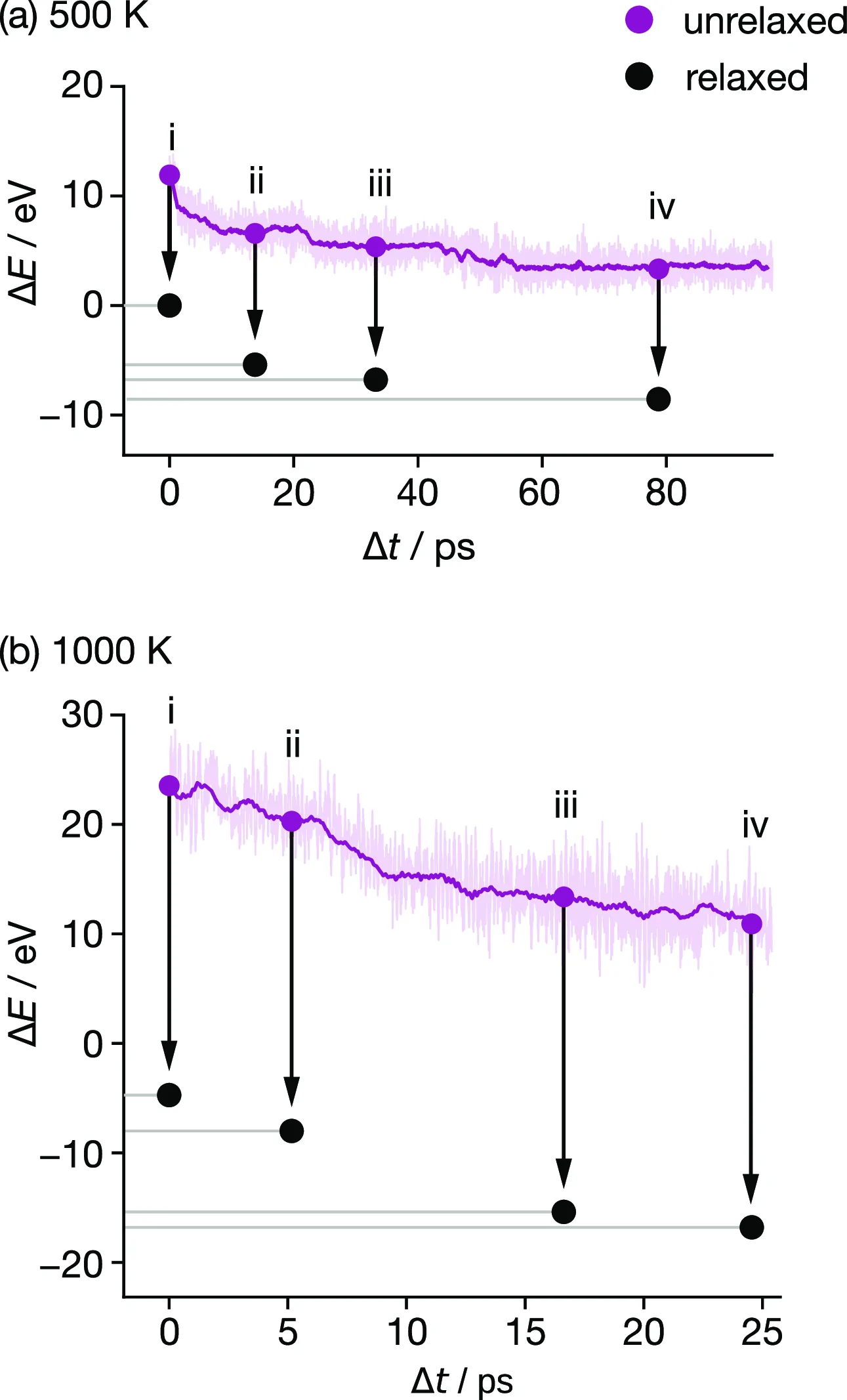
Total energy evolution during AIMD simulations of FeSiO_4_ at (a) 500 K and (b) 1000 K, shown as a moving average. Purple points (i–iv) indicate frames selected for DFT relaxation from each simulation. Black points show the corresponding relaxed energies. All energies are relative to the relaxed unreorganized FeSiO_4_ structure.

Figure 6 shows the calculated Fe–Fe, Fe–Si, and O–O radial distribution functions (RDFs) for all four relaxed frames from the 500 K trajectory and for the final relaxed frame (frame iv) of the 1000 K trajectory. The 500 K trajectory RDFs show substantial loss of structure beyond ∼3 Å, with the disappearance of well-defined peaks indicating progressive disordering. We also observe new peaks appearing at short distances: these shorter Fe–Fe and Fe–Si distances indicate migration of Fe and/or Si from their initial tetrahedral sites. Fe coordination numbers extracted from the relaxed frames confirm this migration: the number of tetrahedrally coordinated Fe falls from 36 (of 36) in frame i to 9 in frame ii of the 500 K trajectory, with migrated Fe occupying predominantly five-coordinate sites (22 of 27).

**Figure 6:**
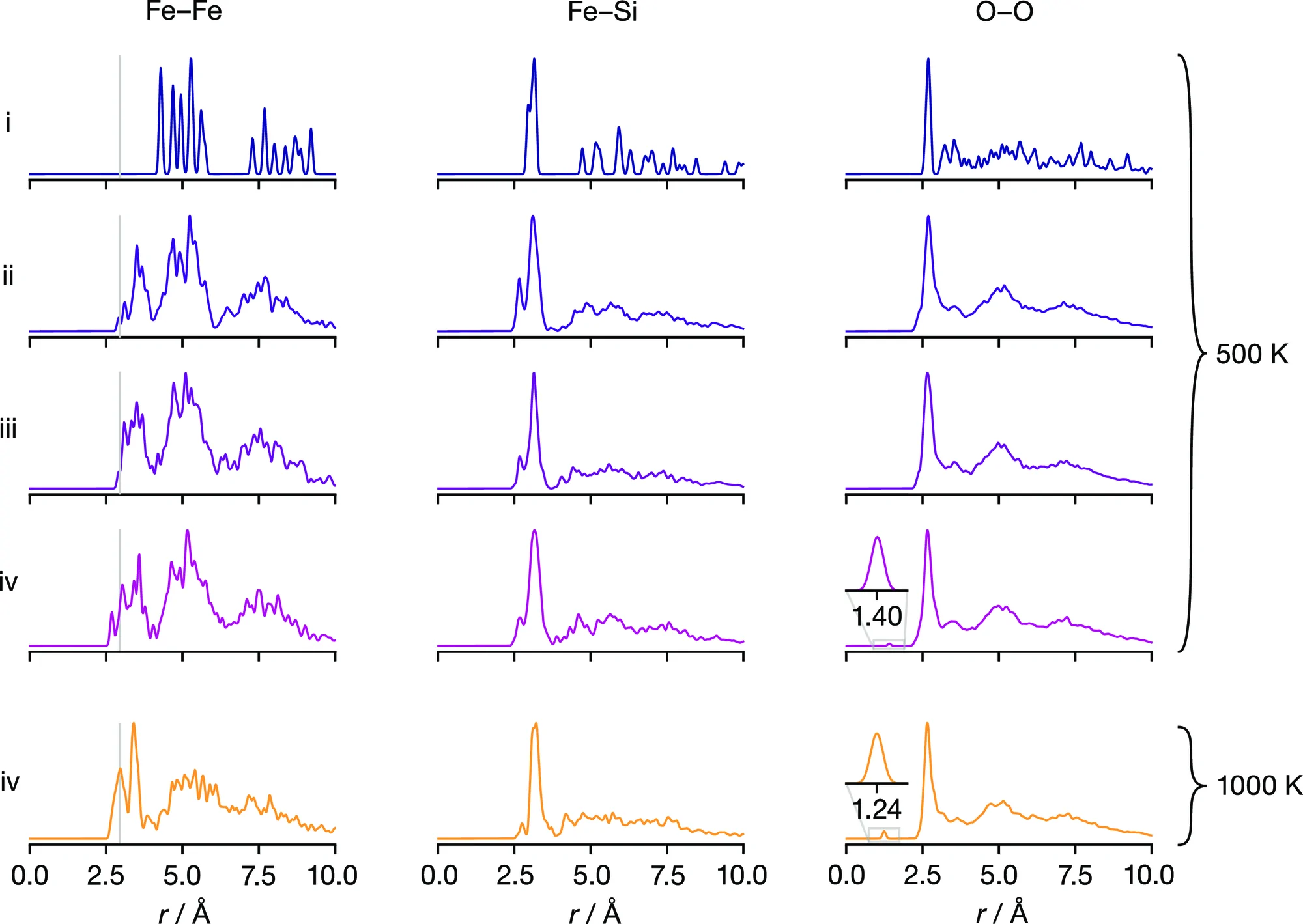
Radial distribution functions for Fe–Fe, Fe–Si, and O–O pairs in FeSiO_4_, from relaxed frames i–iv of the 500 K trajectory and frame iv of the 1000 K trajectory, from the AIMD simulations shown in Figure 5. The gray vertical lines in the Fe–Fe plots correspond to the edge-sharing Fe–Fe distance in Fe_2_O_3_ (2.97 Å).^77^

The appearance of Fe–Fe peaks at short distances (<3.0 Å), compared to the nearest-neighbor distance of 4.3 Å in the starting FeSiO_4_ structure, is somewhat surprising: these Fe were assigned a formal oxidation state of Fe^IV^ in the starting structure and are expected to carry large positive charges, suggesting they should remain well separated. Along the 500 K trajectory, two distinct short Fe–Fe peaks emerge; in frame iv of the 1000 K trajectory, these merge into a single broad peak. These short Fe–Fe distances can be explained by noting that FeSiO_4_ is predicted to be unstable with respect to formation of Fe_2_O_3_ (Section 3.2). In Fe_2_O_3_, edge-sharing FeO_6_ octahedra give an Fe–Fe distance of 2.97 Å;^77^ the merged peak in the most reorganized frame is approximately centered on this distance (gray lines in the Fe−Fe panels of Figure 6). Direct visualization of these relaxed frames shows that the short Fe–Fe contacts arise predominantly from edge-sharing FeO_6_ polyhedra, with additional face-sharing pairs appearing in the most reorganized frames. The emergence of short Fe–Fe distances therefore reflects the formation of Fe_2_O_3_-like structural motifs, consistent with the onset of the predicted decomposition.

A second structural change is revealed by the O–O RDFs: frame iv of each trajectory shows new peaks, at 1.40 Å and 1.24 Å for the 500 and 1000 K simulations, respectively. These features correspond to oxygen dimer formation, confirmed by direct visualization of the atomic positions. The 1.40 Å bond length in frame iv of the 500 K trajectory indicates a peroxide or superoxide species; inspection of the structure reveals this dimer bridges between SiO_4_ and FeO_6_ polyhedra. In contrast, frame iv of the 1000 K trajectory contains three dimers with 1.24 Å bonds that are largely dissociated from the host framework (minimum Fe–O distance of 2.01 Å), characteristic of molecular O_2_. This O_2_-containing frame is also the lowest in energy of all our relaxed frames (Figure 5), indicating that O_2_ formation by local structural rearrangement is energetically favored, consistent with our thermodynamic analysis (Section 3.2).

The short Fe–Fe distances appear well before O–O dimerization: in the 500 K trajectory they are already present in frame ii (13.8 ps), whereas the first peroxide forms at 54.4 ps. This temporal sequence, with Fe migration preceding oxygen dimerization, contrasts with the behavior reported for lithium-rich layered oxides, in which these processes occur concurrently.^9^ The decomposition of FeSiO_4_ thus proceeds through distinct structural steps.

### 3.4 Redox Processes in FeSiO_4_

The formation of oxygen dimers during our AIMD simulations demonstrates that delithiation from LiFeSiO_4_ to FeSiO_4_ involves redox processes that extend beyond a simple Fe^III^/Fe^IV^ couple. To characterize these redox processes, we performed Wannier analysis on each relaxed AIMD frame to assign oxidation states throughout the observed structural transformations. These Wannier-based assignments are consistent with calculated Fe magnetic moments, and with Fe–O ICOBIs and mean bond lengths, which scale systematically with assigned oxidation state (Supporting Information, Figure S3), providing independent validation of the oxidation-state classification.

Figure 7 shows the evolution of Fe and O oxidation-state populations throughout both AIMD trajectories. In the unreorganized FeSiO_4_ structure, all Fe are in the +IV state and all O are in the −II state. By the final frames, substantial reduction of Fe^IV^ to Fe^III^ has occurred, yielding 22% Fe^III^ in frame iv (500 K) and 36% Fe^III^ in frame iv (1000 K). These final frames also show oxidized oxygen species: frame iv (500 K) contains a peroxide dimer (O_2_^II–^) and frame iv (1000 K) contains three molecular O_2_ species (both consistent with the O–O distances described in Section 3.3). This coupled reduction of Fe^IV^ to Fe^III^ and oxidation of O^II–^ can be considered a “ligand-to-metal charge transfer” process,^30,31,78^ in which high-valent Fe centers are reduced by electron transfer from oxygen ligands, while these oxygen anions are oxidized to form O_2_.

**Figure 7:**
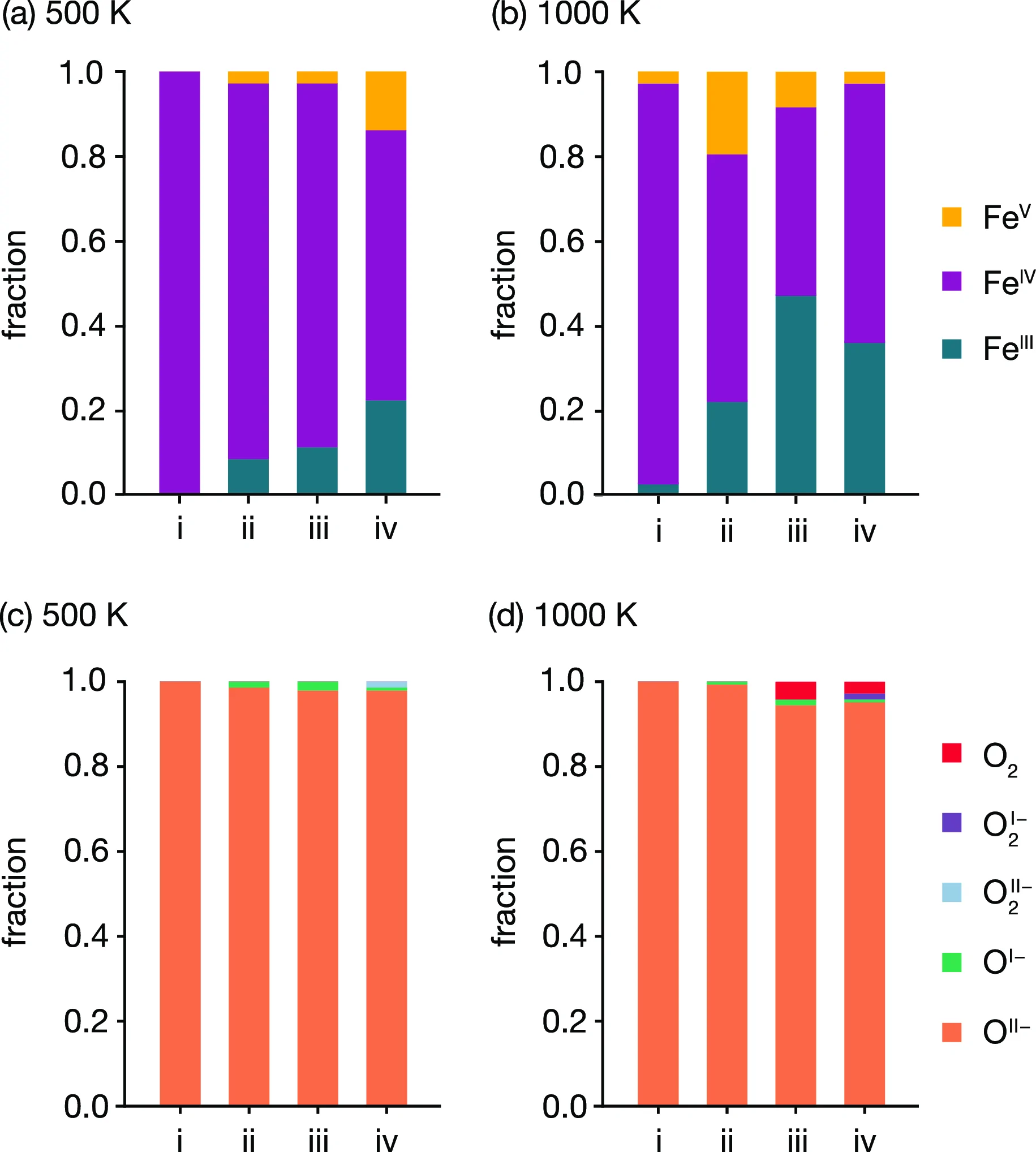
Wannier-assigned oxidation-state populations for (a,b) Fe and (c,d) O in FeSiO_4_, for relaxed frames i–iv from the AIMD trajectories shown in Figure 5. Panels (a,c) refer to the 500 K trajectory; panels (b,d) refer to the 1000 K trajectory.

While the redox behavior of FeSiO_4_ appears relatively straightforward when considering only the initial and final MD frames, the intermediate frames reveal a more complex picture. In both MD trajectories, we observe reduction of Fe^IV^ to Fe^III^ before any oxygen dimerization. This reduction must be balanced by corresponding oxidation elsewhere in the system. O^I–^ species (holes localized on oxygen) provide part of this compensation, but the O^I–^ population is at no point sufficient to account for all the observed Fe^III^. The remainder is provided by oxidation of other Fe^IV^ centers to Fe^V^; this coupled formation of Fe^III^ and Fe^V^ corresponds to disproportionation of Fe^IV^. In the 1000 K trajectory the Fe^V^ population peaks at 7 of 36 Fe in frame ii before falling to 1 of 36 in frame iv as oxygen dimers form, indicating that Fe^V^ is reduced during oxygen dimerization; in the 500 K trajectory, in which only a single peroxide forms, the Fe^V^ population instead increases monotonically, to 5 of 36 in frame iv.

Comparing the Fe coordination environments and Fe oxidation states across both trajectories shows that transition-metal migration largely precedes disproportionation. In the 500 K trajectory, after 13.8 ps, 27 of 36 Fe have left tetrahedral coordination, while only four Fe have changed oxidation state (three Fe^III^, one Fe^V^). Over the remaining 65 ps, only two additional Fe change coordination, whereas disproportionation continues to develop: by 78.7 ps, 13 of 36 Fe have changed oxidation state (eight Fe^III^, five Fe^V^). The same temporal ordering is observed at 1000 K: by frame i, 32 of 36 Fe have already left tetrahedral coordination, while only two have changed oxidation state. Migration therefore occurs while Fe is predominantly Fe^IV^. The migrated Fe mostly occupy new five-coordinate environments rather than octahedral sites: Fe^V^ shows a stronger preference for octahedral coordination (three of five and five of seven in the frames with the largest Fe^V^ populations at 500 and 1000 K, respectively) while Fe^III^ has a lower average coordination.

## 4 Summary and Discussion

Lithium-rich cathode materials offer substantially higher capacities than conventional cathodes but typically suffer from poor cycling stability, associated with molecular O_2_ formation and irreversible structural change at high states of charge.^6,16^ Polyanionic frameworks, such as the silicate ${\left[{SiO}_{4}\right]}^{4-}$ units in Li_2_FeSiO_4_, have been proposed to stabilize these materials against O_2_ formation;^25^ if effective, this protection could allow such lithium-rich cathodes to be reversibly cycled at high capacity.

Here, we have used DFT calculations and AIMD to examine the sequential delithiation of Li_2_FeSiO_4_. Geometry optimizations on Li_2_FeSiO_4_, LiFeSiO_4_, and FeSiO_4_ show that, in the absence of structural reorganization, the delithiation of Li_2_FeSiO_4_ can be characterized as formal Fe^II^ → Fe^III^ → Fe^IV^ based on magnetic moments and Wannier electron counting. The first lithium removal (Li_2_FeSiO_4_ → LiFeSiO_4_) is well described as conventional cationic redox; i.e., Fe^II^ → Fe^III^. The second lithium removal (LiFeSiO_4_ → FeSiO_4_), however, exhibits mixed cationic/anionic redox character: while we formally assign this step as Fe^III^ → Fe^IV^, substantial electron density is also removed from oxygen atoms due to high Fe–O covalency in FeSiO_4_. Because the hole is substantially delocalized onto oxygen, this Fe^IV^ state would not be expected to give a clear Fe^4+^ spectroscopic signature. O K-edge absorption spectra of fully delithiated FeSiO_4_ have been interpreted as Fe^3+^ with a ligand hole rather than Fe^4+^;^40^ this description is equivalent to the formal Fe^IV^ assigned here.

We also find fully delithiated FeSiO_4_ to be both thermodynamically and kinetically unstable. Static DFT calculations predict that FeSiO_4_ is unstable with respect to decomposition into Fe_2_O_3_, SiO_2_, and O_2_, while AIMD simulations predict that the unreorganized structure spontaneously rearranges to form Fe_2_O_3_-like motifs and molecular O_2_, consistent with the end products of the thermodynamically predicted decomposition. This result contradicts the proposal that Si–O covalence prevents formation of molecular O_2_ in silicate cathodes.^25^ Instead, the predicted reorganization and O_2_ formation mirror those reported for lithium-rich layered^9^ and disordered rocksalt materials.^11,14^

Further analysis of the AIMD trajectories resolves this redox process into a sequence of steps. Overall, Fe^IV^ is reduced to Fe^III^ with concomitant oxidation of O^II–^ to form molecular O_2_. This reduction of high-valent transition metals coupled to oxygen oxidation has previously been described as ligand-to-metal charge transfer,^30,31,78^ and our AIMD trajectories provide a concrete atomic-scale example of this process.

Tracking oxidation states through Wannier analysis shows that this is not a simple reduction of Fe^IV^ with coupled O_2_ formation. In our AIMD trajectories, Fe^IV^ disproportionates to Fe^III^ and Fe^V^, with Fe^V^ reaching 7 of 36 Fe in intermediate frames of the 1000 K trajectory, before falling to 1 of 36 as oxygen dimers form, indicating that Fe^V^ is reduced during oxygen dimerization. This instability of Fe^IV^ is consistent with observations in other Fe-containing systems, in which formal Fe^IV^ often exists as a disproportionated Fe^III^/Fe^V^ mixture.^79,80^ Fe^V^ itself has recently been reported as an electrochemically accessible stable species in Li_4_FeSbO_6_.^81^ Unlike in lithium-rich layered systems, in which transition-metal migration and oxygen dimerization occur simultaneously,^9^ we find that in FeSiO_4_, Fe migration occurs first, with disproportionation developing subsequently in the migrated structure and oxygen dimerization later still.

The redox sequence we observe in Li_2_FeSiO_4_—initial Fe^IV^ formation with high Fe–O covalency, followed by Fe migration from tetrahedral sites, disproportionation to Fe^III^ and Fe^V^, and oxygen dimerization with reduction of Fe^V^ as O_2_ forms—incorporates multiple processes that have been proposed as competing explanations for oxygen redox in lithium-rich cathodes, including high-valent metal formation, ligand-to-metal charge transfer, and oxygen dimerization to peroxide and molecular O_2_.^9,11,19,25,30,31,36,37^ Our results suggest these are not competing alternatives but sequential components of a compound process.

A similar compound mechanism has been proposed for Li_2_MnO_3_, a prototypical lithium-rich layered oxide:^38,39^ Mn^4+^ oxidizes to high-valent Mn^6+/7+^ coupled with migration from octahedral to tetrahedral sites, followed by an electrochemically silent step in which Mn reduces back to Mn^4+^ while oxygen oxidizes to form trapped molecular O_2_. Transition-metal migration is identified there as a precursor to O–O dimerization, consistent with the ordering we observe here. The details differ in ways that reflect the different crystal chemistry: in Li_2_MnO_3_, migration to tetrahedral sites enables the high-valent Mn state, whereas we find Fe migrating out of tetrahedral sites while still predominantly Fe^IV^, with disproportionation to Fe^III^ and Fe^V^ developing subsequently in the migrated structure—a step with no counterpart in the proposed Mn mechanisms.

The extent to which transition-metal disproportionation plays a role in other lithium-rich systems remains an open question. If the compound picture holds more generally, the relative prominence of each step would be expected to depend on the specific material’s electronic structure, the accessibility of high-valent states, and the kinetic barriers to metal migration and O–O bond formation. Establishing which of these steps recur across lithium-rich chemistries, and which vary, would determine how far the compound picture extends.

Our predictions for Li_2_FeSiO_4_ are open to direct experimental test. Molecular O_2_ formation could be probed by resonant inelastic X-ray scattering, which has been used to identify trapped O_2_ in lithium-rich oxide cathodes,^10,19^ and by differential electrochemical mass spectrometry, which has been used to detect O_2_ evolved from lithium-rich cathodes during charging.^82^ The predicted evolution of the Fe oxidation states—including Fe^V^ formation—could be followed by Mössbauer spectroscopy, which has previously been used to characterize disproportionated Fe^III^/Fe^V^ in formally Fe^IV^ oxides^79,80^ and has recently been applied operando to lithium-rich layered cathodes.^78^

## Supplementary Material



## Data Availability

The complete raw data set of DFT calculation inputs and outputs is available from the University of Bath Research Data Archive.^83^ A companion repository containing analysis code and the minimal extracted data required to reproduce figures and results is available on GitHub;^84^ this includes scripts for plotting Figures 2, 3 and 5–7 and S1–S3 and for reproducing the Wannier oxidation-state assignments and thermodynamic stability calculations.

## Notes

Justification for assuming the inverse *Pmn*2_1_ polymorph of Li_2_FeSiO_4_ (Section S1); validation of DFT protocol via calculation of the Li_*x*_FeSiO_4_ convex hull and voltage curve (Section S2); details of oxidation-state assignment from Wannier centers, including treatment of dimeric species (Section S3); additional analysis of Fe magnetic moments and Fe–O bonding as a function of Wannier-assigned oxidation state (Figure S3) (PDF)
