# Supplementary material for: Multi-Step Oxygen Redox Mechanism in the Polyanionic Lithium-Rich Cathode Li2FeSiO4
Source: J Am Chem Soc. 2026 Sep 28;148(40):42928–38. doi: 10.1021/jacs.6c02850 (PMC13674281; doi:10.1021/jacs.6c02850)
Supplement: Supplementary file 1 [file ja6c02850_si_001.pdf]

Supporting Information for "Multi-Step Oxygen Redox Mechanism in the Polyanionic Lithium-Rich Cathode Li_2FeSiO_4"

Patrick J. Taylor [1,2,*] Kit McColl [1,2,3] M. Saiful Islam [2,3] and Benjamin J. Morgan [1,2,*]

[1] *Department of Chemistry, University of Bath, Claverton Down, Bath BA2 7AY, United Kingdom*
[2] *The Faraday Institution, Quad One, Harwell Science and Innovation Campus, Didcot OX11 0RA, United Kingdom*
[3] *Department of Materials, University of Oxford, Oxford OX2 6NN, United Kingdom*

S1: On the Assumption of the Inverse $Pmn2_1$ Polymorph of Li_2FeSiO_4

All DFT calculations in this work were performed on the inverse $Pmn2_1$ polymorph of Li_2FeSiO_4, although several other polymorphs have been observed experimentally. We focused on this single polymorph for two reasons. Firstly, we lacked the computational resources to repeat our analyses across all relevant structures. Secondly, we expect the inverse $Pmn2_1$ polymorph to be representative of the experimentally cycled material. X-ray and neutron diffraction data suggest that regardless of which polymorph is initially synthesised, electrochemical cycling transforms the structure to inverse $Pmn2_1$ [1]. DFT calculations further predict that the inverse $Pmn2_1$ polymorph is lowest in energy at full delithiation, providing a thermodynamic rationale for this transformation [2].

To test this assumption, we compared the relative energies of all experimentally observed Li_2MSiO_4 polymorphs (M = Co, Fe, Mn, Ni), computed for Li_xFeSiO_4 at $x = 2$ and $x = 0$, following Ref. [2]. As shown in Fig. S1, the inverse $Pmn2_1$ polymorph is 10 meV/atom above the most stable polymorph at $x = 2$, but is the lowest-energy structure at $x = 0$. This agrees with Ref. [2], although we find that the Pmnb and $P2_1$ polymorphs are only marginally less stable at $x = 0$.

Our results thus support previous experimental and computational studies indicating that the inverse $Pmn2_1$ polymorph forms during extended cycling. We therefore consider this polymorph the most representative single structure for modelling Li_2FeSiO_4.

S2: Convex Hull and Predicted Voltage Curve for Li_xFeSiO_4

The voltage curve for Li_xFeSiO_4 consists of two consecutive plateaus, with a step at $x = 1$. To validate our DFT protocol, we computed a convex hull spanning the $x = 2 \rightarrow x = 0$ composition range and predicted a voltage curve. To generate the convex hull, we enumerated all symmetry-inequivalent lithium-vacancy orderings in the Li_2FeSiO_4 primitive cell and performed DFT relaxations on each configuration. We also randomly generated and relaxed 65 additional configurations in $2\times1\times1$ and $2\times2\times1$ supercells. All configurations were generated using the ICET Python package [3], sampling as evenly as possible across compositional space.

As shown in Fig. S2(a), the only points on the hull occur at $x = 2$, $x = 1$ and $x = 0$, consistent with previous calculations [2] and with the stepped voltage profile reported experimentally [4]. The corresponding voltage curve (Fig. S2(b)) has plateaus at 2.70 V and 4.91 V. The first plateau is within 0.1 V of experimental measurements; the second is predicted at a higher voltage than observed experimentally ($\sim$ 4.5 V). The origin of this discrepancy is unclear; we note that the measured high-voltage capacity may not correspond to equilibrium delithiation to unreorganised $FeSiO_4$, with parasitic electrolyte reactions reported at these voltages and temperatures [4].

S3: Assigning Oxidation States from Wannier Centres

Oxidation states were assigned from maximally localised Wannier functions by associating each Wannier centre with its nearest atom, yielding an integer electron count for each atom. Here we provide additional details on obtaining the Wannier functions and assigning oxidation states for dimeric species such as O_2.

We used the selected columns of the density matrix (SCDM) method to generate initial projections for all Wannier bases. The relevant density matrix is the real-space variant expressed in the position basis, with **k**-resolved components of the form

$$P_{ij}^{\boldsymbol{k}} = \sum_n f(\epsilon_{n\boldsymbol{k}}) \psi_{n\boldsymbol{k}}(\boldsymbol{r}_i) \psi_{n\boldsymbol{k}}^*(\boldsymbol{r}_j). \tag{S1}$$

The "occupation function" $f(\epsilon_{n\boldsymbol{k}})$ is effectively a free parameter in the SCDM method: it does not encode Kohn–Sham occupation numbers, but instead selects and weights the Bloch states from which Wannier functions are derived. For oxidation-state assignments, we set

$$f(\epsilon_{n\boldsymbol{k}}) = \begin{cases} 1 & \text{if } \epsilon_{n\boldsymbol{k}} \leq E_\mathrm{F} \\ 0 & \text{if } \epsilon_{n\boldsymbol{k}} > E_\mathrm{F} \end{cases}, \tag{S2}$$

thereby building the density matrix solely from the occupied Bloch states beneath the Fermi level E_F.

We followed a similar approach when Wannierising the lowest-energy unoccupied states for Li_2FeSiO_4 and

* patrick.joseph.taylor@gmail.com, b.j.morgan@bath.ac.uk

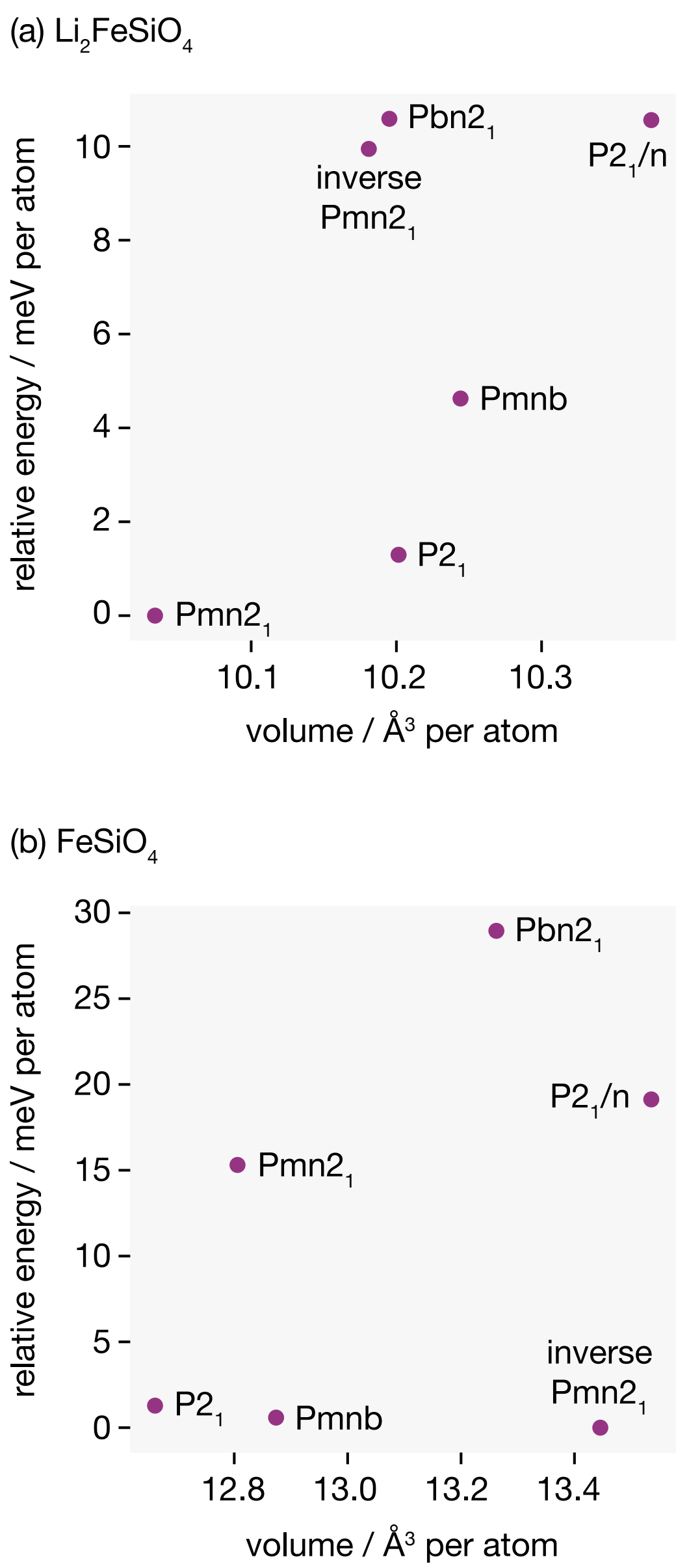

FIG. S1. Energies and volumes for various polymorphs (labelled by space group) of (a) Li_2FeSiO_4 and (b) $FeSiO_4$, in both cases given relative to the lowest-energy polymorph.

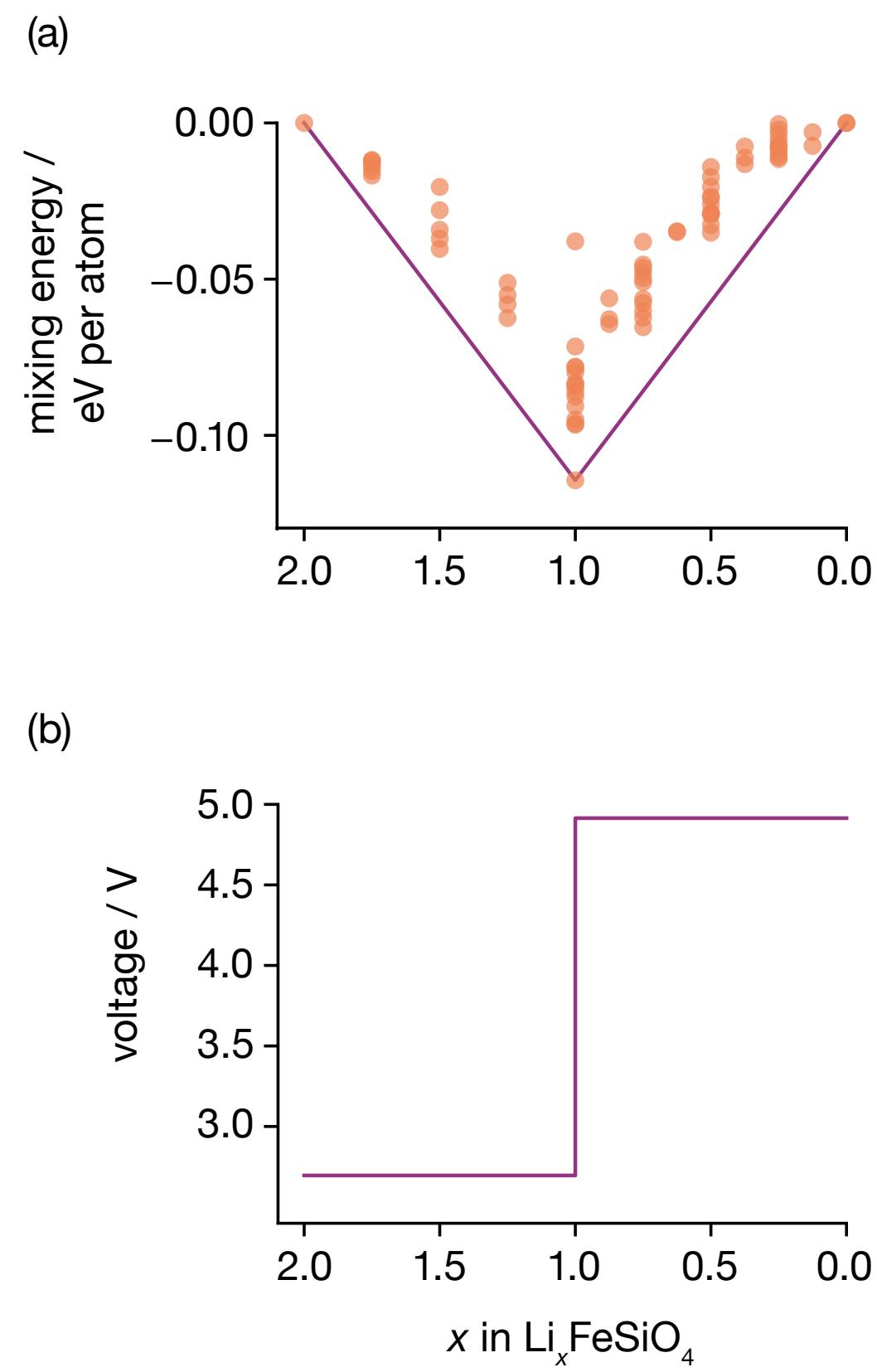

FIG. S2. (a) The convex hull and (b) voltage curve predicted by our DFT calculations for the delithiation of Li_2FeSiO_4.

$FeSiO_4$, selecting bands within a strict energy window to exclude other bands from the density matrix. For the first and last relaxed frames of our 1000 K AIMD trajectory, and for the lowest-energy unoccupied states in $LiFeSiO_4$, we adjusted $f(\epsilon_{n\boldsymbol{k}})$ to account for energetic entanglement with adjacent bands. In these cases, we smoothed the energy window cutoffs to include minor contributions from the adjacent states. The specific occupation functions are provided in the VASP input files in the accompanying dataset [5].

Assigning oxidation states to individual atoms is straightforward: each Wannier centre is associated with its nearest atom, yielding an integer electron count. For dimeric species such as O_2, however, this approach requires additional consideration. In some cases it works well—for example, two O^0 atoms within ~ 1.22 Å of one another can be identified as an oxygen molecule. However, the covalent nature of O–O bonds can complicate assignments: Wannier centres from bonding states may lie approximately equidistant from both oxygen atoms. If these are assigned unevenly, an O_2 molecule might appear as $O^{I-} + O^{I+}$ rather than two O^0. To avoid this ambiguity, we assign oxidation states to dimeric species by summing the Wannier centres associated with both oxygen atoms.

We apply this correction when the O–O ICOBI ≥ 0.95 (bond order ~ 1 or greater); such pairs are counted as dimers and their Wannier centres summed. This ensures correct identification regardless of the positioning of interatomic Wannier centres: $O^{I-} + O^{I+}$ yields an overall oxidation state of 0.

As additional validation of these assignments, we compared Fe magnetic moments, Fe–O ICOBIs, and Fe–O bond lengths across the Wannier-assigned oxidation states; Fig. S3 shows the magnetic-moment and ICOBI distributions. Each Fe in each of the eight relaxed frames contributes one observation, classified by its oxidation state in that frame; the ICOBI and bond-length values are means over that centre's Fe–O bonds, taken from the LOBSTER ICOBI output so that both refer to the same

bonds. Mean absolute magnetic moments are $4.00\,\mu_B$, $3.30\,\mu_B$, and $2.44\,\mu_B$, mean Fe–O ICOBIs 0.28, 0.43, and 0.53, and mean Fe–O bond lengths 1.94 Å, 1.86 Å, and 1.83 Å, for Fe^{III}, Fe^{IV}, and Fe^{V}, respectively.

All three quantities vary monotonically with assigned oxidation state, with the magnetic moments falling in near-uniform steps of $\sim 0.8\,\mu_B$. The distributions are broad and overlap between adjacent oxidation states, most notably in the bond lengths, but the Wannier assignments, magnetic moments, ICOBIs, and bond lengths are mutually consistent, with each higher formal oxidation state corresponding to shorter, more covalent Fe–O bonding.

[1] A. R. Armstrong, N. Kuganathan, M. S. Islam, and P. G. Bruce, Structure and lithium transport pathways in Li_2FeSiO_4 cathodes for lithium batteries, J. Am. Chem. Soc. **133**, 13031 (2011).

[2] A. Saracibar, A. Van der Ven, and M. E. Arroyo-de Dompablo, Crystal structure, energetics, and electrochemistry of Li_2FeSiO_4 polymorphs from first principles calculations, Chem. Mater. **24**, 495 (2012).

[3] M. Ångqvist, W. A. Muñoz, J. M. Rahm, E. Fransson, C. Durniak, P. Rozyczko, T. H. Rod, and P. Erhart, ICET – A Python library for constructing and sampling alloy cluster expansions, Adv. Theory Simul. **2**, 1900015 (2019).

[4] T. Masese, C. Tassel, Y. Orikasa, Y. Koyama, H. Arai, N. Hayashi, J. Kim, T. Mori, K. Yamamoto, Y. Kobayashi, H. Kageyama, Z. Ogumi, and Y. Uchimoto, Crystal structural changes and charge compensation mechanism during two lithium extraction/insertion between Li_2FeSiO_4 and $FeSiO_4$, J. Phys. Chem. C **119**, 10206 (2015).

[5] P. J. Taylor, K. McColl, M. S. Islam, and B. J. Morgan, Dataset for "Multi-Step Oxygen Redox Mechanism in the Polyanionic Lithium-Rich Cathode Li_2FeSiO_4", University of Bath Research Data Archive, `https://doi.org/10.15125/BATH-01647` (2026).

[6] M. Allen, D. Poggiali, K. Whitaker, T. R. Marshall, J. van Langen, and R. A. Kievit, Raincloud plots: A multi-platform tool for robust data visualization, Wellcome Open Res. **4**, 63 (2021).

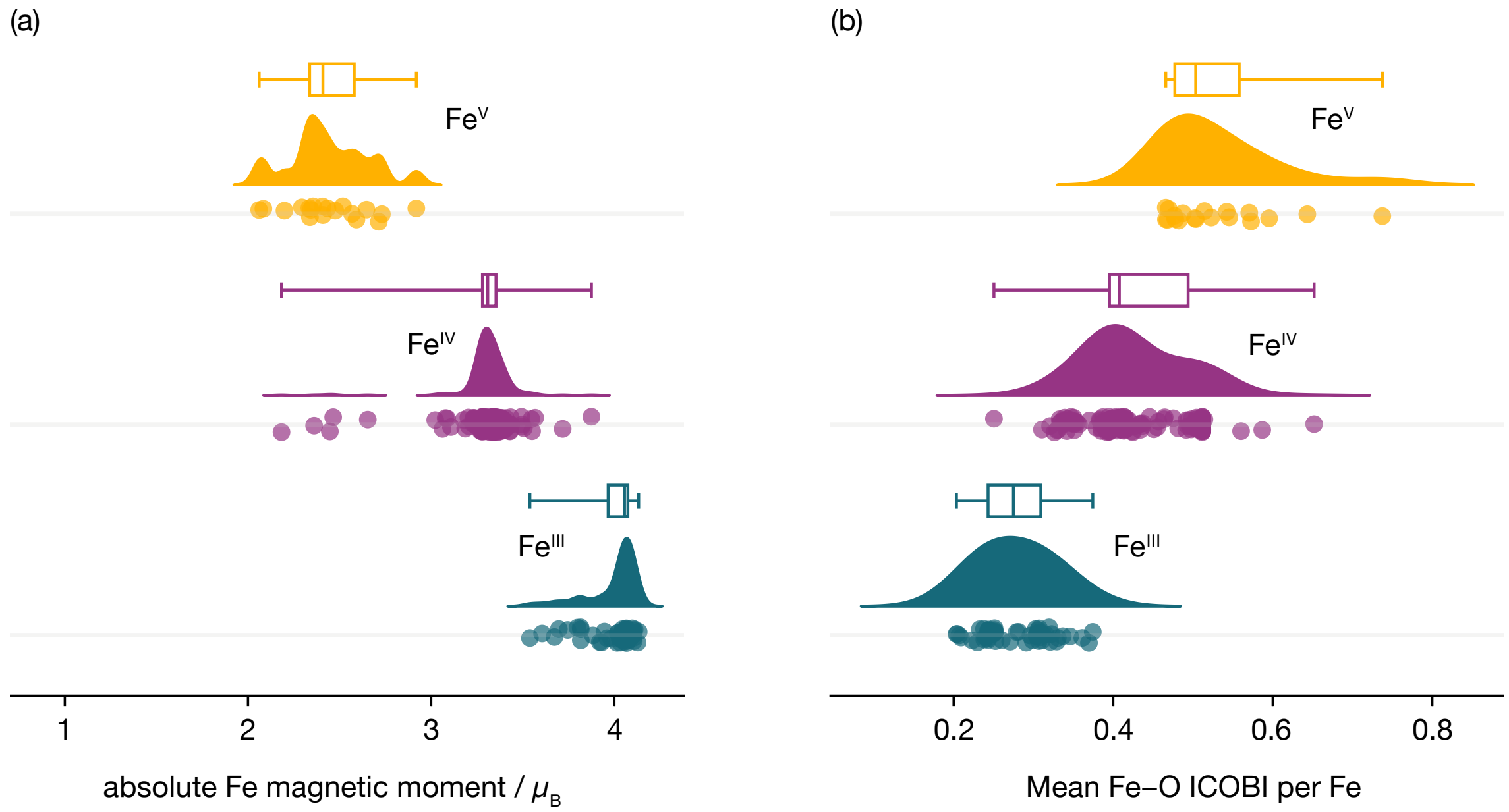

FIG. S3. Raincloud plots [6] showing the distributions of (a) absolute Fe magnetic moments and (b) mean Fe–O ICOBIs, per Fe, in $FeSiO_4$, for all Wannier-assigned iron oxidation states. Boxes indicate the interquartile range (25th–75th percentile) with the median marked; whiskers extend to the full data range.
